# Longer Gestation among Children Born Full Term Influences Cognitive and Motor Development

**DOI:** 10.1371/journal.pone.0113758

**Published:** 2014-11-25

**Authors:** Emma V. Espel, Laura M. Glynn, Curt A. Sandman, Elysia Poggi Davis

**Affiliations:** 1 Department of Psychology, University of Denver, Denver, Colorado, United States of America; 2 Department of Psychology, Crean College of Health and Behavioral Sciences, Chapman University, Orange, California, United States of America; 3 Department of Psychiatry and Human Behavior, College of Medicine, University of California Irvine, Irvine, California, United States of America; University of Tuebingen Medical School, Germany

## Abstract

Children born preterm show persisting impairments in cognitive functioning, school achievement, and brain development. Most research has focused on implications of birth prior to 37 gestational weeks; however, the fetal central nervous system continues to make fundamental changes throughout gestation. Longer gestation is associated with reduced morbidity and mortality even among infants born during the period clinically defined as full term (37–41 gestational weeks). The implications of shortened gestation among term infants for neurodevelopment are poorly understood. The present study prospectively evaluates 232 mothers and their full term infants (50.4% male infants) at three time points across the first postnatal year. We evaluate the association between gestational length and cognitive and motor development. Infants included in the study were full term (born between 37 and 41 weeks gestation). The present study uses the combination of Last Menstrual Period (LMP) and early ultrasound for accurate gestational dating. Hierarchical Linear Regression analyses revealed that longer gestational length is associated with higher scores on the Bayley scales of mental and motor development at 3, 6 and 12 months of age after considering socio-demographic, pregnancy, and infant-level covariates. Findings were identical using revised categories of early, term, and late term proposed by the Working Group for Defining Term Pregnancy. Our findings indicate that longer gestation, even among term infants, benefits both cognitive and motor development.

## Introduction

Recent findings question the conventional 37-week cut off for preterm birth. A large literature indicates that children born preterm show persisting impairments in cognitive functioning, school achievement and brain development. Preterm birth is associated with altered hemispheric connections [1], loss of gray/white matter [2], [3], disrupted myelination, and abnormal cortical folding [4]. Cognitive impairments are present even among late preterm infants. For example, infants born between 34 and 37 weeks' gestation (late preterm) are more likely to have lower mental and psychomotor developmental scores than those born full term [5]–[7]. Even though research has focused on the consequences of birth prior to 37 weeks gestation, fetal brain development undergoes fundamental changes throughout gestation. We investigate here if there are significant relations between gestational age at birth and neurodevelopment among infants born full term.

Longer gestation is associated with reduced morbidity and mortality even among infants born during the period clinically defined as full term (37–41 weeks gestational age) [8]–[11]. For example, infants born between 37 and 38 weeks experience greater morbidity, including diseases such as respiratory distress syndrome, hospital admissions within the first five years of life, and limiting longstanding illness by 5 years of age, than those born between 39 weeks 0 days and 41 weeks 6 days [12], [13]. Additionally, birth at a younger gestational age predicts short stature and elevated blood pressure among young men who were born at term [14]. Because of the evidence that gestational length is negatively associated with physical health outcomes even when analyses are restricted to those born post 37 weeks, recent efforts have been made to move away from traditionally dichotomized categories of preterm and full term [11], [15]. The American Medical Association Workgroup for Defining Term Pregnancy suggested that early term be defined as delivery between 37–38 weeks gestation, term as delivery between 39–40 weeks gestation, late term as 41 weeks gestation and post-term as delivery after 41 weeks [11].

Little is known about the consequences of variation in gestational length among term infants for cognitive functioning and brain development. Rapid changes in the organization of the fetal brain continue into late gestation [16], [17], and shortened gestation is associated with reduced grey matter volume and higher network efficiency among 6–10 year old children who were full term at birth [18], [19]. Moreover, 6.5 year-old children born early term (37–38 gestational weeks) score significantly lower on intelligence quotient (IQ) tests than their counterparts born between 39 and 41 weeks [20]. Similarly, Noble and colleagues [21] report that gestational length among children born at term is positively associated with reading and math achievement scores in third grade. Executive function (i.e., reaction time accuracy and speed) at age six also is predicted by gestation length among children born after 37 weeks' gestation [22]. These studies provide evidence that gestational length influences cognitive development even among children born after the clinical cutoff of 37 gestational weeks. One limitation of these studies is their retrospective design. Only one study has examined the relation between gestational length and cognitive outcomes among full term infants within the first year of life. In a large Chilean sample, Rose and colleagues [23] found that longer gestational length is associated with increased cognitive and psychomotor development scores beyond the effect of potential confounds such as socioeconomic status and home environment. This study provides evidence that gestational length among term infants is associated with cognitive development during infancy. One limitation of this study is the reliance solely on maternal report of last menstrual period for gestational dating. Early ultrasound, before 22 gestational weeks, consistently has been found to be a more accurate predictor of delivery date than maternal report of menstrual cycle, with menstrual dating underestimating delivery date [24], [25]. Precise dating is essential to determine the relation between gestational age and outcomes among infants born full-term.

The present study prospectively evaluates mother-infant pairs across the first postnatal year and uses the American Congress of Obstetricians and Gynecologists (ACOG) standard, a combination of Last Menstrual Period (LMP) and early ultrasound for accurate gestational dating. We evaluate the association between gestational length and cognitive and motor development among full term infants assessed longitudinally at three time points during the first postnatal year.

## Method

### Participants

Study participants included 232 mothers and their full term infants participating in a longitudinal study evaluating the role of early experiences on infant development. Women with singleton pregnancies less than 16 weeks gestational age (GA) were recruited from obstetric clinics in Southern California and followed longitudinally through 12 months postpartum. Women were eligible for participation in this study if they were English-speaking, non-smokers, over the age of 18, did not take steroid mediation, and for whom there was no evidence for drug or alcohol use during pregnancy. Twenty-three (8.9%) participants were excluded due to preterm birth, and 4 (1.5%) were removed due to post-term birth. The 232 infants (50.4% male) included in the current study were full term at birth– born between 37 weeks 0 days and 41 weeks 6 days gestation (*M* GA = 39.46 weeks, SD = 1.06 weeks, *M* weight = 3418.10 g, *SD* = 419.71 g). All infants were normal and healthy at birth, and they had a median 5-minute Apgar score of 9 (range = 8–10). Additional descriptive information for the study sample is shown in Table 1.

**Table 1:** Demographic Information for the Study Sample.

*Maternal Demographics*	
Maternal age at delivery [*M* (SD)]	29.76(5.56)
Cohabiting with baby's father (%)	88.4
Birth order (% first live birth)	45.7
*Education Highest Attainment (%)*	
High school or equivalent	12.7
Some college/college	68.4
Graduate degree	15.4
*Annual Household Income*	
*Obstetric Risk*	.32(.56)
$0–$30,000	24
$30,001–$60,000	23.6
$60,001–$100,000	31.1
Over $100,000	21.4
*Race/ethnicity (%)*	
Non-Hispanic White	43.1
Latina	27.6
Asian	8.6
Other	20.7

### Ethics

Mothers gave written informed consent for themselves and their child for all aspects of the protocol. This study protocol (HS #2002-2441) was approved by the Institutional Review Board for Protection of Human Subjects at the University of California, Irvine.

### Measures

#### Birth Outcome

Gestational Age at Birth (GAB) was determined with standard (high-accuracy) ACOG guidelines (2009) using the last menstrual period and an ultrasound prior to 20 weeks gestational age. Additionally, parity, birth weight, and Apgar scores were recorded from the medical chart review. An extensive structured medical interview was conducted by a research nurse at each prenatal visit to assess current and past maternal health and pregnancy related complications. Additionally, maternal and infant medical records were reviewed to assess pregnancy complications and birth outcome. These two sources were used to derive a well-established index characterizing prenatal obstetric risk and considered in covariate analyses (Hobel, 1982).

#### Infant Development

Infant development was assessed at 3, 6, and 12 months postpartum using the Bayley Scales of Infant Development, 2^nd^ ed. (BSID-II). Examiners were trained by a clinician with over 15 years of experience with the BSID and were directly supervised by a clinical psychologist. Videotaped assessments were reviewed monthly. Interrater reliability, calculated on 20% of the assessments at each age, was 95% at 3 and 6 months and 93% at 12 months. The BSID is a standardized developmental assessment [26]. Measures of development were obtained at each age using the Mental Development Index (MDI) and the Psychomotor Development Index (PDI).

Reliability, calculated using coefficient alpha based on the normalization samples on the BSID were between.83 and.92 for the ages assessed here. Conventional scoring of the BSID created composite MDI and PDI scaled scores by summing the total number of items achieved, corrected for basal effects. The raw scores were converted to scaled scores by reference to a developmental table. Mean scaled scores for the MDI and PDI are presented in Table 2. Consistent with expectations, intercorrelations between MDI scores across 3, 6, and 12 months showed modest stability (r's = .23 to.38, p's<.01), as do those between PDI scores across 3, 6, and 12 month assessments (r's = .23 to.47, p's<.01).

**Table 2:** Infant BSID Score (Mean and Standard Deviation).

	Infant age in months		
	*3*	*6*	*12*
Psychomotor Development Index	93.91(8.51)	98.59(12.24)	97.58(16.43)
Mental Development Index	93.44(6.48)	97.34(8.59)	93.84(10.81)

Note. BSID = Bayley Scales of Infant Development, 2^nd^ ed. (BSID-II). Scores are mean indexed scores.

### Data Analysis

Correlations, t-tests, and Analysis of Variance (ANOVA) were used when appropriate to identify socio-demographic (i.e., race/ethnicity, cohabitation status, maternal education, household income), pregnancy-related (i.e., prenatal medical risk), and infant (i.e., birth order, sex, birth weight percentile, infant age from conception) variables that might influence infant cognitive or motor development. Race/ethnicity, cohabitation status, maternal education, household income obstetric risk, birth order, birth weight percentile and infant sex were included as covariates in the model if they were associated with infant development. The factors associated with infant development (BSID scores) with a *p* value of.10 or less were included as covariates. Variables associated with MDI (ethnicity, parity, maternal age at delivery, obstetric risk, birth weight percentile) or PDI (ethnicity) in preliminary analyses were modeled as covariates at the ages they were associated with the corresponding outcomes in regression analyses. Age from conception at the time of testing (3, 6, or 12 months) was not correlated with chronological age from birth at testing and thus, age from conception at BSID assessment did not account for study findings.

Hierarchical Linear Regression analyses were conducted to identify the association between gestational length and postnatal cognitive and motor development at 3, 6, and 12 months after adjusting for covariates. Covariates were entered in the first step, and GAB was entered second into the model.

In addition to continuous analyses, we applied the American Medical Association Workgroup for Defining Term Pregnancy criteria (early term, term, and late term gestation length categorizations) and evaluated the associations with mental and psychomotor development using Univariate Analysis of Variance with Covariates (ANCOVAs). The same covariates, described above, were used for both the Hierarchical Linear Regression and the ANCOVA models.

## Results

### Gestational length and Infant Development

Hierarchical regression analyses reveal that longer gestational length is associated with higher mental and psychomotor development at each infant age beyond the effects of the covariates (see Table 3). Among infants born full term, longer gestation is associated with higher MDI scores at 3 months (*F*(7, 177) = 3.95, *p*<.001, *R^2^* = .14), 6 months (*F*(7, 167) = 2.45, *p*<.05, *R^2^* = .11), and 12 months (*F*(7, 153) = 2.84, *p*<.01, *R^2^* = .12). Gestational length also is associated with elevated PDI scores at 3 months (*F*(4, 180) = 3.03, *p*<.05, *R^2^* = .06), 6 months (*F*(4, 169) = 5.47, *p*<.001, *R^2^* = .12), and 12 months (*F*(4, 156) = 3.36, *p*<.05, *R^2^* = .08).

**Table 3:** Main Effects and Interactions for Relationship Domains Predicting Psychosocial Outcomes.

	Unstandardized Regression Coefficients for Outcomes Measured at Month:								
	3			6			12		
Predictors	*b*	S.E.	β	*b*	S.E.	β	*b*	S.E.	β
***Cognitive Development***									
Ethnicity^a^									
Latina	−2.73†	1.46	−.19	−1.83	2.07	−.06	−6.07*	2.77	−.25
White	−3.00*	1.34	−.23	−.68	1.95	.002	−6.65†	2.54	−.31
Asian	−2.00	1.91	−.09	−.84	2.58	−.04	−3.25	3.41	−.09
Birth Order^b^	.87	.96	.07	1.73	1.36	.08	−1.00	1.79	−.05
Maternal Age	−.11	.09	−.09	.05	.12	.02	.06	.16	.03
Obstetric Risk	−.01	.82	−.001	−1.20	1.08	−.07	−3.22*	1.43	−.18
Birth Weight Percentile				.05*	.02				
GAB (95% Confidence Interval)	1.72*** (.86–2.58)	.43	.28	1.54* (.38–2.70)	.59	.24	1.99* (.45–3.51)	.77	.20
***Psychomotor Development***									
Ethnicity									
Latina	.03	1.96	.001	−6.58*	2.82	−.25	−6.57	4.16	−.18
White	−1.12	1.79	−.07	−1.93	2.63	−.08	−2.62	3.82	−.08
Asian	−.02	2.55	−.001	−4.00	3.53	−.10	−2.84	5.12	−.05
GAB (95% Confidence Interval)	1.90** (.77–3.04)	.58	.24	2.94*** (1.37–4.51)	.80	.27	3.79** (1.52–6.05)	1.15	−.25

^a^Ethnicity is dummy coded for Latina, White, and Asian.

^b^Birth order is dummy coded so a score of 1 indicates first pregnancy.

† *p*<.10.

*p<.05.

**p<.01.

***p<.001.

**Figure 1:**
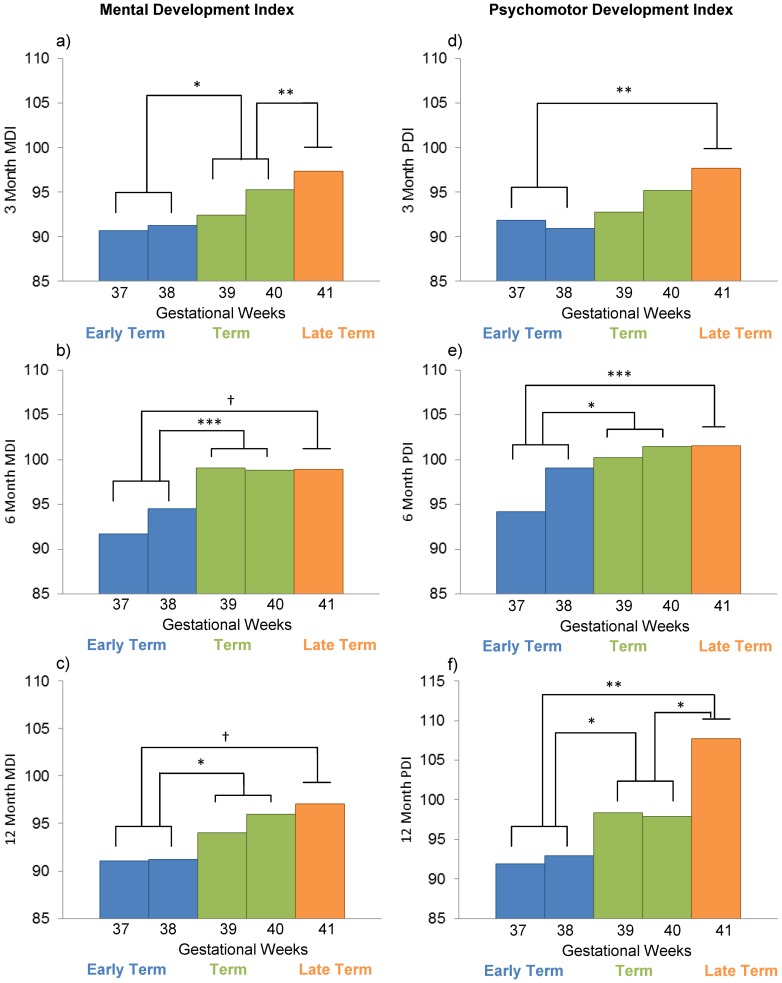
Gestational length predicts Mental and Psychomotor Development. Gestation length predicts mental and psychomotor development at 3, 6, and 12, months follow-up. Bars represent weeks of gestation, colors represent gestation groups identified by the Workgroup for Redefining term pregnancy. Post-hoc pairwise comparisons using Fisher's Least Significant Difference were conducted between Early Term, Term, and Late Term groups (not by week) and are denoted. ^†^ *p* = .08 *p<.05 **p<.01 ***p<.001.

Using the Working Group for Defining Term Pregnancy revised categories 27% of our sample was early term, 56% term, and 6.6% late term. ANCOVAs reveal that group differences in mental development are evident at 3 months (*F*(2, 177) = 7.15, *p*<.001) and 6 months (*F*(2, 167) = 7.23, *p*<.001), with a trend at 12 months (*F*(2, 153) = 2.87, *p* = .06). As presented in Figure 1 a–c, term infants score lower on mental development indices at three months than those born late term. Infants born early term scored lower than those born at term or late term on mental development at 3 months and 6 months, and lower than those born at term at 12 months on mental development. There also are group differences in psychomotor development at each assessment age. At 3 months, psychomotor development is lower for infants born early term compared to those born late term (*F*(2, 179) = 4.01, *p*<.05)(Figures 1d–f). Early term infants exhibit lower psychomotor development scores than term and late term infants at both 6 (*F*(2, 168) = 6.69, *p*<.01) and 12 (*F*(2, 155) = 5.32, *p*<.01) months of age. Term infants had lower psychomotor development scores than late term infants at 12 months.

## Discussion

The Fetal Programming hypothesis posits that in utero experiences exert consequences for health and development that persist across the lifespan. Gestational length is affected by *intrauterine* experiences, such as exposure to stress, that affect both fetal maturation and timing of delivery [27]–[29]. The present study documents that even modest variations in gestational length among full-term infants exert programming influences on cognitive and motor development. Our data demonstrate that among a low-risk group of healthy full term infants, longer gestation is associated with improved mental and motor development at 3, 6, and 12 months of age. These findings call into question the conventional cut off of 37 gestational weeks for preterm birth, and support the argument that gestational length should be regarded as a continuum of development throughout pregnancy.

Accumulating evidence documents the importance of gestational length among term infants on a range of outcomes. The American Medical Association Workgroup for Defining Term Pregnancy recently has argued the importance of redefining term delivery based on evidence that both morbidity and mortality are increased among infants who are born at 37–38 gestational weeks compared to infants born at 39–41 gestational weeks. Their report suggests that the consequences of gestational length be considered among term as well as preterm infants. We report that the benefits of longer gestation for full term infants extend beyond physical health outcomes to include significant neurodevelopmental benefits. This association was significant when gestational age was analyzed continuously or categorically using the proposed redefinition of term delivery. Further, associations remained after considering socio-demographic factors, and obstetric factors known to be associated with neurodevelopment, as well as chronological age from conception at time of assessment.

These present findings complement a recent and important study [23] in a large Chilean sample that reported longer gestation is associated with higher BSID mental and motor scores at one year of age. Our study extends these findings by (i) precisely characterizing gestational length using the “gold-standard” based on early ultrasound and LMP and (ii) applying a prospective longitudinal design with three assessments during the first postnatal year to characterize the relations between gestation length and infant outcomes across the first year. The present findings, in conjunction with the study by Rose and colleagues, add evidence to previous literature linking gestational length among children born at term to IQ [20], executive functioning [22] and school performance [21] in childhood. By prospectively characterizing cognitive and motor development during the first prenatal year the present study provides evidence that associations between gestational length and cognitive development are not likely due to postnatal confounding variables.

Rapid changes in the development and organization of the fetal brain occur throughout the entire course of gestation extending into the term period [16], [17]. During late pregnancy, fetal development includes gyri formation, neuronal differentiation, dendritic arborization, axonal elongation, synapse formation and collateralization, and myelination [16], [17]. Linear increases in total gray matter volume of 1.4% per week are seen from 29 to 41 gestational weeks [30] and approximately 50% of the increase in cortical volume occurs between 34 and 40 gestational weeks [31], [32]. Because of the massive developmental changes that occur late in gestation, the fetal brain is susceptible to influences such as those associated with shortened gestation. Consistent with this argument, we recently have shown that shortened gestation among children who were born full term is associated with gray matter development, primarily in temporal regions [33] and reduced neural network efficiency [19].

It is plausible that the associations between gestational age at birth and child neurodevelopment reported here are due to prenatal influences on processes that mature the last few weeks of pregnancy. Our findings in this low-risk healthy sample suggest that prenatal experiences (e.g., normative exposure to maternal and placental hormones) benefit fetal brain development [18], [34]. The advantage of longer gestation for neurodevelopment is arguably a result of prenatal influences that benefit the developing fetal nervous system. Infants born at an earlier gestational age additionally are more likely to experience adverse events such as hypoxia that have neurological consequences. Alternatively, the association between length of gestation and cognitive development may be a byproduct of gestational exposures, such as placental CRH that influence both gestational length and the construction of the fetal nervous system [35]–[37].

### Strengths and Limitations

Strengths of this investigation include the prospective longitudinal design and the precise assessment of gestational length. Infants in this sample are healthy, and have not experienced complications that pre-term infants often do, such as mechanical ventilation, intraventricular hemorrhage, or periventricular leukomalacia. The effects of gestational length are present after considering potential confounds of obstetric risk, ethnicity, maternal age at delivery, breastfeeding, and parity. A limitation of the present study is that infants were assessed only through one year of age and thus, it is not known if this association persists into childhood. In the present study, global measures of mental and motor development were implemented. Future research might evaluate specific functions that may be more or less susceptible to shortened gestation. Further, this study does not identify the mechanisms underlying the association between shortened gestation and developmental outcomes.

### Implications

These data support the redefinition of term delivery as proposed by the American Medical Association Workgroup for Defining Term Pregnancy. The implications for clinical practice are clear and argue against the relatively common practice of elective inductions or cesarean sections prior to 39 gestational weeks. Early term inductions occurred in 8% of births in the United States in 2006 [38]. Further, rates of cesarean section are as high as 32% of all births in the US and in other countries such as Brazil, where rates are as high as 80% among those delivering in private hospitals [39]. Given the large number of scheduled deliveries modest decreases in cognitive and motor development associated with shortened gestation among term infants may have profound implications at a population level. As with climate change where a difference of a few degrees can alter the landscape, shortening gestational length by 1–2 weeks may shift the distribution of IQ at the population level and may increase rates of developmental disabilities and compromise intellectual capital. Consistent with this possibility, modest differences in IQ are associated with substantive differences in educational attainment and occupation. For example, college graduates differ from non-graduates by just a few IQ points and managers have a mean IQ that is just 3 points higher than their employees [40]. Additionally, understanding the impact of gestational length influence methods used for the appropriate assessment of infant development. These data suggest that when early screening for developmental delays is implemented with young infants, gestational length should be considered for full term as well as preterm infants. Finally, it is possible that the modest cognitive and motor delays associated with shortened gestation establish a developmental trajectory that may lead impairments that become exacerbated over time.

Developmental processes occurring during the fetal period shape the development of physiological systems with consequences for birth outcomes as well as physical and mental health across the life span. Consistent with the Fetal Programming hypothesis, we show with a prospective design that longer gestation benefits cognitive and motor development among healthy full term infants. These findings have clear implications for clinical decisions.
